# Care during ART scale-up: surviving the HIV epidemic in Ethiopia

**DOI:** 10.1057/s41292-022-00283-7

**Published:** 2022-10-03

**Authors:** Makoto Nishi

**Affiliations:** Hiroshima University, 1-7-1, Kagamiyama, Higashi-Hiroshima City, Hiroshima, 7398521 Japan

**Keywords:** Antiretroviral therapy, Care, Ethiopia, HIV, Pharmaceuticalization, Treatment as prevention

## Abstract

Over the last decades, there has been a worldwide rise of new technologies for controlling the HIV epidemic by expanding antiretroviral medicines. This article examines how the pharmaceutical-driven model of public health, which emerged as a byproduct of antiretroviral treatment (ART) scale-up in Ethiopia, interplayed with local forms of actions, engagements, and voices through which suffering inflicted by the epidemic was cared for. Through the eyes of an Ethiopian woman with HIV, this article illustrates how the increasing emphasis on ART facilitated the defunding of some community-based care practices. Moreover, it rendered the realities of precarious life with HIV invisible in the landscape of therapeutic citizenship. However, for Ethiopians, ART scale-up unfolded amid multiple forms of HIV care practices and relationships that endured stigma, alienation, and uncertainty before and after ART. The experience of surviving the HIV epidemic in Ethiopia provides a vital premise upon which claims of meaningful care are made, and ways to otherwise develop healthcare actions and engagements are sought.

## Introduction

The last decades have seen the rise of new technologies to control the HIV epidemic by expanding the use of antiretroviral medicines (ARVs). This article explores how some aspects of pharmaceuticalization unfolded within and profoundly transformed the domain of HIV care in Ethiopia. Through the eyes of an Ethiopian woman with HIV, the article illustrates the interplay between the increasing emphasis on antiretroviral treatment (ART) and local forms of actions and engagements through which the suffering inflicted by the epidemic is cared for.

Pharmaceuticalization^1^ is defined as the concept of capturing what happens “when pills are introduced to manage complex health problems,” particularly in social settings of limited health and welfare resources (Nguyen 2015, p. 60). This article aims to address the process through which ART rollout has altered the actions and engagements of those affected by HIV (Nguyen 2010; Whyte 2014b; McKay 2018). It also illuminates how the process transformed the modes of HIV intervention and how these embraced or rejected sufferers’ voices (Biehl 2007).

I define care as encompassing a range of engagements and evoking normative claims to address the problems of those affected by the HIV epidemic. Care, on one hand, is embedded in situated practices (McKay 2018). Ramah McKay describes care as encapsulating “all the material, social, epistemological, and medical work that accompanies pharmaceuticals” (2018, p. 16). However, care also evokes normative claims. McKay explains that the conceptualizations of care “not only draw attention to health inequities and serve as calls for action; they also reflect normative political claims about what the state is or should be” and “serve to index caring subjects and subjectivities in ways that are raced, classed, and gendered” (p. 16).

In terms of situated engagements and normative claims, the rollout of the free nationwide ART program in Ethiopia profoundly altered the forms of HIV care. I argue that this process has largely been facilitated by “new prevention technologies” (Nguyen 2015, p. 50), which accompanied the scale-up of HIV treatment in South Africa (Mahajan 2018), Brazil (Biehl 2007), and elsewhere in the Global South. Such technologies include treatment-as-prevention (TasP), pre-exposure prophylaxis (PrEP), prevention of mother-to-child transmission (PMTCT), and voluntary male circumcision. Manjari Mahajan argues that these interventions are “resolutely biomedical” (2018, p. 149). In contrast to many older prevention strategies, including behavior change programs and broad-based social measures, new techniques are “animated by logics of calculable efficacy” (Mahajan 2018, p. 149).

Among these techniques, TasP has played the most significant role in Africa (Nguyen 2015; Mahajan 2018), including Ethiopia. The advancement of the TasP strategy has been based on the assumption that the HIV epidemic in a given population may be eliminated by achieving the universal administration of ARVs. When properly adhered to, ARVs significantly reduce the risk of viral transmission because they reduce the amount of target virus circulating in bodily fluids (Eisinger et al. 2019). The aggressive expansion of ART to reach all individuals with HIV and upholding medication adherence among those on ART are considered vital for a successful TasP intervention.

Epidemiologists have invested considerable efforts to predict the efficacy of this strategy by constructing mathematical models (Granich et al. 2009; Cohen and Gay 2010). These findings were supported by a large-scale trial involving multiple countries (Cohen et al. 2011) and other studies involving human subjects (Smith et al. 2011). Moreover, the outlook that the universal treatment strategy may swiftly and cost-effectively end the HIV epidemic in the Global South has helped consolidate financial assistance from wealthy nations and private funds, facilitating the global expansion of ART during the last decade.

How the scale-up of ART and the accompanying process of pharmaceuticalization altered the landscape of HIV care in Africa has been the subject of multiple medical anthropological inquiries (Nguyen et al. 2011; Moyer 2015; Kenworthy et al. 2018). Susan Whyte noted that when ART became widely available in Uganda, stories of fear and death were replaced by “tales of miraculous resurrection through the power of medicine” (2014a, p. 13). However, she argued that life with ART was inherently characterized by “chanciness” or dependence on an uncertain event, occurrence, or relationship (Whyte 2014a, p. 20). In the city of Kisumu, Kenya, the survival of individuals with HIV often depended on contingent care networks. Ruth Prince argued that the “moral economy of survival” was medicalized by the scaling-up of ART programs by highlighting medication adherence while rendering the realities of life—particularly persistent hunger—invisible (2012, p. 548).

The insecurity of life in Africa has partly been reinforced by the “defunding” of primary healthcare services under the shadow of ART scale-up (Kenworthy et al. 2018, p. 965). The increasingly technocratic nature of HIV interventions, coupled with austerity policies that dominate international funding agencies, have rendered public health personnel in Mozambique chronically and increasingly overworked and overburdened (Pfeiffer and Chapman 2015, 2019). Moreover, the existing forms of non-pharmaceutical HIV care are often redefined to fit the technocratic scheme. Since the early stages of the HIV epidemic, home-based care (HBC) has been practiced in some African countries as a community-level response. When HBC was incorporated into the national HIV program in Mozambique in the early 2000s, the program became more technically comprehensive, but the practice was narrowly redefined to be clinically oriented work (Kalofonos 2014).

In what follows, this study examines how the technocratic scheme that accompanied the ART scale-up rendered the suffering of some people invisible in the landscape of public health interventions in Ethiopia. Moreover, it explores how some effects of the new technology in HIV prevention and care interplayed with local forms of actions and engagements through which the voices of sufferers were heard and their problems were addressed.

The following section provides a brief account of the ART scale-up that profoundly altered the landscape of HIV care in Ethiopia. It is primarily based upon government reports and data provided by funding agencies. The remaining sections closely examine some of the effects of pharmaceuticalization on life during the epidemic, mainly based on my interviews with a woman named Meseret Gebre,^2^ who lived in Welkite, a town in the southern part of the Ethiopian highland. The interviews were conducted during my visits to Ethiopia to conduct ethnographic fieldwork between September 2007 and October 2015.^3^ During our sessions, she shared stories concerning the lives of her own and some fellow HIV-positive Ethiopians, part of which are recounted in this article. We visited places such as health facilities in and around Welkite town and households in her home village to trace her trajectory. We also conducted group meetings and a questionnaire survey with members of an association of HIV-positive people, of which Meseret was the representative. Some key figures in HIV care and advocacy groups and government health bureaus were also interviewed. I used Amharic, an Ethiopian Semitic language spoken as lingua franca in the country, as the medium of communication.

### Scaling-up ART in Ethiopia

The Ethiopian government has made significant efforts to expand ART over the last decades. The official ART program was introduced in Ethiopia in July 2003. Initially, each patient was charged 300–700 ETB (or 35–82 USD at the time) per month, which was a significant barrier to wide drug dissemination. Urged by the WHO and UNAIDS, which jointly launched the initiative to expand ART to 3 million people worldwide by the end of 2005, the government decided to provide ART free of charge for its poorer citizens (HAPCO 2006).

The free ART program was launched in January 2005. However, this was hindered by another barrier. Patients were required to visit the local *kebele* office (the lowest level of government administration in Ethiopia) to obtain a “poverty certificate” for free ART (Kloos et al. 2007). Considering the extremely high level of stigma against HIV/AIDS in the country during those days, it is understandable that patients found it unbearable to expose their status to the *kebele* officials, who might not keep the information private. In the same year, the government decided to provide free ART to all its citizens, regardless of their economic status.^4^

The rollout of ART in Ethiopia coincided with a period of aggressive investment in the public health sector when Dr. Tedros Adhanom (who later became the Director-General of the WHO) served as the health minister between 2005 and 2012. HIV units were established in renovated and newly established government health centers throughout the country, with staff members conducting screenings, consulting patients, disseminating ARVs, and maintaining patient records. In 2014, ART was provided at 1,047 health facilities, of which 849 were public health centers (HAPCO 2014). ART coverage in Ethiopia increased from 3% in 2005 to 56% in 2014 and to 78% in 2020.^5^ AIDS-related deaths and HIV incidence decreased during the same period.^6^ The free ART program is largely financed by the Global Fund to Fight AIDS, Tuberculosis and Malaria (hereafter, the Global Fund).^7^ Ethiopia has been among the largest recipients of the fund in the last decade.^8^

Alongside the ART scale-up, self-help associations among HIV-positive people have proliferated in Ethiopia. Some began operations during the 1990s, representing crucial aspects of HIV care work and relationships in Ethiopia while providing social and material support, including peer counseling, HBC, and food and monetary assistance for their members. Between 2004 and 2010, many such associations were established countrywide and encouraged via moral and financial support from transnational NGOs and funding agencies.

The national universal ART program, coupled with non-pharmaceutical programs conducted by self-help associations, once seemed to comprise an ideal state–civil society partnership in HIV care. However, as a result of dwindling HIV spending and a shift in government policy toward NGOs, it became increasingly evident that non-pharmaceutical care activities were underfunded and marginalized. Between July 2011 and June 2012, 125 million USD were spent on HIV treatment and care programs, of which 69 million (55%) were spent on ART, according to data compiled by the Ethiopian government (HAPCO 2013). In contrast, only 1.4 million (1.1%) went to HBC, a pillar of self-help association activities.^9^ In Mozambique, HBC was incorporated into the national program and was redefined to fit a scheme that narrowly defined care (Kalofonos 2014). In Ethiopia, it was not redefined but was defunded and deserted.

As their vital care resources shrank, self-help associations were left without access to alternative financial or other forms of support. In 2009, the government of Ethiopia issued a proclamation that severely restricted the activities and resource access of non-governmental organizations (NGOs), including the associations of HIV-positive people.^10^ Furthermore, the government made it extremely difficult for international NGOs to assist the activities of local NGOs (DAG 2013).^11^ As a result, a handful of international institutions, including the Global Fund, became the only sources of substantial financial support for HIV-positive people in Ethiopia. The government became virtually the sole channel through which the associations could access such support.

However, for Ethiopians, the national ART program unfolded within a multiplicity of HIV care practices and relationships, some of which took their form prior to the arrival of ARVs and others that emerged alongside the free ART program. In the following sections, I offer a closer look at some effects of the ART scale-up on HIV care in Ethiopia.

### Meseret

I first met Meseret in September 2007 in Welkite, the capital of the Gurage Zone, located 150 km southwest of Addis Ababa. The Gurage Zone is an administrative unit in the Southern Region of Ethiopia. I was planning to commence a new research project concerning local responses to the HIV epidemic. During a visit to the office of a local NGO engaged in HIV interventions, I saw a poster that promoted HIV screening. The poster included a photograph of a woman with HIV and her message: “HIV screening made it possible for me to know myself and consider what to do for my life.”

The woman on the poster was Meseret. She was in her 1920s and served as a representative of a small association of HIV-positive people living in and around the town. Our first interview took place at her office. I told her that I saw the poster and was impressed by the message. She said, “Let me tell you what I had encountered. Some people who saw my poster hanging on a wall tsk-tsked [to show pity], saying, ‘This woman should have been dead.’” Free ART was available in Ethiopia at that time, but some continued to perceive AIDS as fatal. However, she did not seem embarrassed by her story. “Thanks to AIDS, I reached where I am now. Otherwise, I was an ordinary rural woman,” she explained during the same interview.

It was only later that I learned what she had gone through before she arrived there. She was born in a remote village 70 km away from Welkite. In her teens, she married an ex-military man. In 1998, shortly after giving birth to a baby boy, the war erupted against Eritrea.^12^ Her husband re-enlisted in the armed forces and was dispatched to the front. He returned sick, and both he and Meseret tested HIV-positive. Similar to other African countries at the time, HIV screening was available at local hospitals and clinics, but treatment was unavailable. Her husband died in 2001. Like many African widows who had lost their husbands to AIDS, Meseret’s status in the village was vulnerable (Geissler and Prince 2010). Her in-laws became increasingly hostile toward her in an attempt to drive her away from her late husband’s land. Recalling those days, she said, “I was in despair at that time. It seemed to me that everyone with HIV would quickly die.” ARVs were virtually inaccessible in Ethiopia during that time, and most people with HIV remained silent and isolated. However, in Addis Ababa, connections among HIV-positive people were already forming.

### Silence

Established in 1997 in Addis Ababa, the Mekdim Ethiopia National Association (hereafter, Mekdim) claims to be the first association of HIV-positive people in Ethiopia. Mengistu Zemene, one of the founding members of the Mekdim association, worked as a police officer in Addis Ababa. He lost his job in 1991 when the Ethiopian Peoples’ Revolutionary Democratic Front (EPRDF, the political coalition that governed Ethiopia until 2019) took control of the country. The following year, he tested HIV-positive. He sought support from the Medical Missionaries of Mary, a religious institute of the Catholic Church. They had established the Counseling and Social Service Center (CSSC) in Addis Ababa in September 1992 to provide support to HIV-positive people and AIDS patients. They offered Mengistu successive counseling sessions, medical treatment, and material support. This led to Mengistu’s physical and mental recovery, after which he decided to help other HIV-positive persons. The CSSC employed him as a social worker and offered him training opportunities in Ethiopia and abroad.

In 1993, after returning from Uganda, where he spent a month visiting institutions engaged in HIV support activities, he established two separate peer support groups with HIV-positive friends. One group met every Saturday at the CSSC. The other was called Gabriel Mahber (Saint Gabriel’s society). *Mahber* is a popular form of voluntary association among the lay followers of the Ethiopian Orthodox Church. Each *mahber* is associated with one of the Christian patron saints; Saint Gabriel was the patron saint for Mengistu’s *mahber*. Members of a *mahber*, who are often close friends, have monthly meetings in one of their homes where they share a meal and engage in discussions. Gabriel Mahber was a small group with eight members, including Mengistu. He wished to use this culturally familiar setting to create a space where HIV-positive persons could provide moral support to one another, especially given their need to cope with a society that perceived their illness as a sign of impending unspeakable death.

A few years later, when Mengistu decided to establish the first association of openly HIV-positive people, his colleagues did not enthusiastically support his ideas. Although Gabriel Mahber was an informal gathering that did not involve any public activity, Mengistu wanted the new association to be legally registered and “more open to the public” (interview, August 17, 2015). However, many of his colleagues considered the idea of becoming openly HIV-positive unacceptable. Although the name of the new association (the Mekdim Ethiopia National Association) did not imply an association with HIV/AIDS, only a handful of people volunteered to serve as formal founders. The name of the association was derived from the Amharic word *meqdim,* which means “prologue.” This word comes from the Amharic verb *qeddeme*, which means “to go ahead.” Unfortunately, it was not easy for HIV-positive individuals in Ethiopia at the time to think about moving ahead.

Meanwhile, Meseret decided to leave her home province for the holy spring on Entoto mountain, which lies on the northern margin of Addis Ababa. Some followers of the Ethiopian Orthodox Church believe that holy springs have the power to cure their illnesses, including AIDS (Guevara 2006). Testimonies of the miraculous healing of the disease by holy water circulate in the local media (Berhanu 2006). Kate Pfizenmaier (2015), who participated in a community health project in Gondar, a city in northern Ethiopia, noted that the tradition offered a unique opportunity to reach marginal populations for HIV education and care because “even people from the most remote villages travel to holy water sites.”^13^

The holy spring at Entoto mountain is preserved by the priests of the Kidist Maryam Church, which was built in 1882 by Menelik II, who later became the Emperor of Ethiopia. Thousands of people with HIV live in shacks on the mountainside. The shacks are owned by local proprietors and rented out to those who seek refuge and spiritual relief. Recalling her arrival, Meseret informed me, “When I got there, no one spoke. But there were so many people who were like that” (Interview, August 8, 2015). She meant that she had met many people living with the virus, but no one spoke openly about their HIV status or AIDS experience. However, Meseret avoided using “HIV” and “AIDS,” perhaps because these had been unspeakable words during her days in Entoto.

The people there avoided talking about the disease as if the silence was a part of the cure they sought. Meseret described a woman who lived in the room next to her: “She did not tell me what she was like, although I told her,” meaning that the woman did not tell Meseret about her HIV status, although Meseret disclosed her own. However, the woman asked Meseret to go to the spring and bring holy water. Meseret had no idea how she could procure the water. The woman explained that she should find the big hall by the holy spring and enter it when they called for the “members of the number two” and that she could receive the water there. Meseret did not ask her what the “number two” was, but she went to the spring and waited for the call. “And then, when they called for the members of the number two, a crowd of people went in, went in, and went in, and the hall was pretty full,” Meseret recalled. Inside the hall, she saw an older woman standing beside her; Meseret asked her if all those people were “like that” (meaning HIV-positive). “My child,” answered the woman, “all these people are like that.” The words struck Meseret. “Immediately, I was relieved. My anxiety, immediately, was gone, was soothed.” She learned that she was not alone, and she somehow felt connected to all the people in the hall that day.

After staying at Entoto mountain for several months, she realized that she was running out of money. She had to pay a considerable rent to the owner of the shack she lived in. One of her neighbors on the mountain suggested that she visit an association called the Tesfa Goh Ethiopia Association (hereafter, Tesfa Goh). She traveled to Addis Ababa to visit the association’s office, where she met a man named Dereje. He asked whether he could help her, but she did not know what to say. He sat down, told her that he was also “like her,” and urged her to speak. She showed him her medical certificate. After reading the certificate, he remained quiet for a while and then left the room. She heard laughter outside the room and thought that they were mocking her for some reason. Dereje returned, gave her some advice, and indicated that they could offer her a means of income. She promised to return another day, but she did not because she was annoyed by the apparent ridicule.

### Takeoff

Dereje was a history teacher when he was diagnosed with HIV infection. He quit his job and joined the community at the Entoto mountain before securing a paid position at Tesfa Goh. Established in 1998, the Tesfa Goh was one of the first associations of openly HIV-positive people in Ethiopia. By the time he met Meseret, Dereje had been one of the most active HIV peer counselors in Addis Ababa (interview, January 1, 2015).

Meseret later learned why Dereje laughed at her when they first met. The problem was with the diagnosis written on her medical certificate. It stated that she had *yezemenu beshita*, meaning the “disease of the time” or the “modern disease.” Ethiopians gave HIV/AIDS this name when it was still considered unspeakable. It is understandable that Dereje and his colleagues, who were already the leading HIV advocates in the country, found it funny (if not outrageous) to have encountered such an obsolete category on an official medical certificate.

As one of the pioneering associations of HIV-positive people in the country, Tesfa Goh attracted international support. During this time, the global movement of HIV-positive people gained momentum. Every international development aid agency operating in Ethiopia provided generous financial support for HIV interventions. Members of the Tesfa Goh association toured the country to conduct advocacy campaigns and established regional branches. Dereje became the head of the association’s Addis Ababa branch and traveled abroad to participate in conferences and capacity-building courses in Kenya, Uganda, Thailand, and elsewhere. Meseret heard Dereje proclaim while recalling the early days of their activities, “We came out during the time when we were seen as *chiraq*. We used to crow like birds.” *Chiraq* is an Amharic word that refers to an imaginary monstrous creature that eats humans; crowed like birds refers to the fact that they not only spoke loudly about HIV, but their voices were heard throughout the country at conferences, on radio, and TV broadcasts.

Meseret left Entoto and returned to Gunchire, a small town located between her village and Welkite. She joined the AIDS awareness campaign organized by the district health office and began to talk to people about the disease while visiting marketplaces, schools, churches, and mosques in the district. In May 2005, Meseret and her colleagues established the Fana Association of HIV-positive people (hereafter, the Fana), the first association of its kind in that area. Later, when she was elected to represent the association, she moved to Welkite, the site of the office.

During the same month, members of the Tesfa Goh association visited the Gurage Zone for an advocacy campaign, with Gunchire as one of their destinations. They remembered Meseret and asked her to participate in the campaign. After Tesfa Goh’s members and Meseret toured several places in the Gurage Zone, they decided to invite her to a national conference of HIV-positive people being held the following month in Nazret. Now renamed Adama, the city is located 100 km southeast of Addis Ababa on the highway to Djibouti; it is one of the largest commercial centers in Ethiopia.

The conference took place over 11 days, starting on June 27, 2005, at the Rift Valley Hotel, which was considered the “fanciest” hotel in the city. Upon arriving at the hotel, she asked herself, “Did I win the DV lottery?” (The DV lottery stands for the Diversity Immigrant Visa Program, allowing thousands of Ethiopians to migrate to the United States every year.) The place was so modern that she wondered whether she was in America. She was astonished by the room equipped with western-style bathrooms and a large TV. She was also surprised that the participants were served breakfast, coffee, lunch, and dinner without being asked to pay.

She remembered that several Ugandans had led the training course. “In the first session, we were asked to tell where we were from, what we liked, and what we disliked. So I said I was from Gurage, I did not like lies, and I liked *kitfo*,” explained Meseret (*Kitfo* is a Gurage cuisine made from chopped raw beef and seasoned with copious butter and spices). Her answer was translated into English so that the Ugandans could understand it. They appreciated her unusual responses and shifted their focus on her. During the sessions, they repeatedly referred to her as the “little girl from Gurage.”

The conference attended by Meseret was officially titled “Leadership and Advocacy Training Course.” It was jointly organized by the AIDS Support Organization (TASO), the famous Ugandan NGO that pioneered care and support programs for people with HIV, and ActionAid Ethiopia, the Ethiopian division of ActionAid International, based in Johannesburg. These NGOs were among the major supporters of HIV advocacy in Ethiopia at the time and provided moral and material support to the emerging movement. Conferences and training courses offered by these organizations gave the leaders of HIV-positive groups in Ethiopia the opportunity to network, discuss their initiatives, and integrate the individual groups into a larger movement. In October 2004, 18 associations in Addis Ababa and other major regional centers created the first national network of associations of HIV-positive people. The networking body was initially named the Association of Ethiopians Living with HIV/AIDS but was later renamed “Network of Networks of HIV Positives in Ethiopia” or NEP+. The network’s membership proliferated, accounting for 60 members in 2008 (NEP+ n.d.) and 450 in 2013 (NEP+ 2013).

In November 2008, NEP+ signed a grant agreement with the Global Fund to implement a project that would cost $26.6 million by its completion in June 2015.^14^ The project provided financial support to participating associations for conducting essential care and support activities, such as HBC, income generation activities, and nutritional support. NEP+’s access to this enormous fund cemented its status as the focal point of the HIV movement in Ethiopia. More than 100 associations, including Tesfa Goh, Mekdim, and Fana, participated in implementing the project (NEP+ 2013).

### Life

“If I were not hesitant at that time, I could have reached some place by now. Sometimes, it is better to be daring than to be hesitant,” Meseret said, recalling 2005, when she was exposed to the quickly expanding national HIV movement (Interview, December 29, 2014). When I added, “You could be living in America by now,” she promptly replied, “Yes, I could. Really.” Meseret knew that quite a few leaders of the early HIV movement in her country had already moved to America. She said she was “hesitant” as she avoided socializing with other participants in the “Leadership and Advocacy Training Course” in June 2005. She quickly returned to her room as soon as the day’s sessions ended.

By the time of her exposure to the national movement, the free ART program had been launched across the country, endowing survivors of the epidemic with another chance to live. They were also invited to an emerging form of therapeutic citizenship, which was very different from the local space of cultural refuge—the one Meseret encountered in the Entoto mountain—where patients silently recognized each other’s suffering. However, Meseret’s choice, which she described as a result of her hesitance rather than her conscience, kept her away from the larger network that could take her to America. Instead, she found herself in a position closer to those who continued to suffer.

After moving to Welkite, Meseret faced financial hardship. Although the government promised to cover the association’s staff salaries, the wages were below sustenance levels and were not regularly paid. She quickly exhausted the small amount of savings that she had accumulated in Gunchire. She also had a difficult time finding affordable housing. Although landlords did not refuse to rent a room because of her HIV status, she once had to move out of a room when its owner suddenly increased the rent price without warning. Another owner asked her to leave because the room needed whitewashing. Her hardship continued until she married a man in Welkite who had a stable income and owned a house.

For individuals with HIV in Africa, access to ART meant a transition from “social death” to living with HIV as a chronic condition (Robins 2005; Russell and Seeley 2010). In some cases, their struggle to regain control of their lives meets a multitude of adversities (Twebaze and Whyte 2014). In Ethiopia, multiple burdens, including social isolation, economic destitution, and comorbidity, characterize the lives of Fana members. In August 2013, Fana had 221 members, 153 of whom were females.^15^ This represented a relatively small portion of the HIV-positive population in the town, which was probably much larger.^16^ In August 2013, I proposed to conduct a questionnaire survey among Fana members to illuminate some features of their problems to Meseret. Fifty individuals were interviewed. Most respondents were either employed in an unstable job (14 day laborers and 7 petty traders) or unemployed (13). Among the 37 female respondents, many were single (4 never married, 10 divorced, and 15 widowed), making them socially and economically vulnerable. Forty-one respondents reported one or more comorbid health problems, and 35 reported that these problems affected their lives either significantly or to a certain extent. They continued to face difficulties despite being on ART and adhering to their medication regimens; 48 of the respondents reported that they had already started ART, and 41 said that they had taken their medicine as prescribed during the previous week.

This concentration of individuals with multiple burdens among Fana members was remarkable, considering that HIV prevalence was probably higher among the rich than the poor in Ethiopia’s general population.^17^ “It is only the poor who come out to join the Fana association,” explained Meseret, “while the rich stay at home.” It could also be said that a localized practice of “triage” (Biehl 2004, p. 109) was at work in this Ethiopian town. Two major health institutions served the population of Welkite: the public health center located in the town and the Catholic hospital located in a neighboring district. Both had on-site HIV units that provided HIV screening, counseling, and ART services. Both units employed a nurse, a pharmacist, a case manager, and an ART support worker. They worked closely with Fana and referred some of their clients, particularly those with social, economic, or other problems that were not necessarily medical, to the association. While this practice helped those ART units stay focused on their primary mandate, which was to manage medication and keep track of adherence, other burdens and life complications were handled by the association.

Further, the following section highlights that Fana was increasingly treated with institutionalized indifference as it had been deprived of already scarce resources to manage its weighty responsibilities. As the TasP-based interventions took hold in Ethiopia, Meseret’s association witnessed a series of practices through which the humanitarian project was steadily swallowed into the sphere of institutionalized indifference.

### Indifference

In 2013, Meseret heard a rumor that financial support for Fana’s HBC activities would soon be terminated. Fana conducted HBC activities for its members for several years; it had 17 volunteer HBC workers and 158 clients in August 2013. The Global Fund provided financial support to such HBC activities in Ethiopia, covering a small transport allowance for each HBC worker. However, apart from this allowance, they did not receive regular official remuneration for their services. Workers visited each client once a week, attending to the needs of the client and their households, including bathing, cleaning, and preparing food. They also monitored the clients’ living conditions, listened to their problems, gave advice, and, if necessary, referred them to a health center. However, some of the clients had issues that were far too serious for the HBC workers to address, as illustrated by the cases of two clients who passed away in 2013.

Bezawit [pseudonym] was one of Fana’s HBC clients. She was working for a local farm, but after giving birth to a child, she developed cancer and was bedridden. She was married to a day laborer, and they rented a hut in a village outside Welkite because they could not afford a room in the town. HBC workers had to walk for an hour to reach Bezawit’s location. They helped her by washing her clothes and cleaning her room but could do little to mitigate her intense pain due to terminal cancer. She could not eat, and the idea of leaving her young child tormented her. When she died, Fana members washed her body before her neighbors buried her.

Welde [pseudonym] was another HBC client who lived alone. He was unable to earn money because he had tuberculosis and was chronically ill. He often moved in search of affordable housing. When he became severely sick, the Fana members tried to help him by collecting money. Because the association did not have a budget to fund his hospitalization, they donated their own money and solicited donations from townspeople. When he died, Fana members sent for his family, but the family denied ever knowing him. His body was taken by the municipal office and buried.

In July 2014, the year following Bezawit and Welde’s deaths, financial support for Fana and other associations’ HBC activities was suspended. One of my informants, whose name I do not cite here, told me that this decision was based on the assumption that most individuals no longer needed HBC support. In the past, many AIDS patients were confined to their beds and required someone to care for them. However, with the advent of widespread ART, fewer people were bedridden. This assumption indicates that individuals such as Bezawit and Welde, who suffered from serious health problems that could not be treated with ARVs alone, were already made invisible in the broader landscape of therapeutic citizenship in Ethiopia.

What happened to the Fana association was similar to Kenneth Maes’s study on the moral economy of community health volunteers in Addis Ababa (Maes 2016; Maes and Kalofonos 2013). HIV intervention programs conducted in the city often relied on local volunteers who were virtually unpaid for the care they provided to their clients.^18^ Maes argued that despite good intentions, using unpaid workers could not be sustainable and that many people remained vulnerable to poverty, unemployment, and poor health.^19^ The health volunteers interviewed by Maes often felt powerless because they did not have adequate resources to address their clients’ problems. Moreover, devoting time and labor to the work that did not generate income to sustain the lives of their own families often became the source of frustration (Maes 2016).

In August 2015, I held a group meeting with some members of the Fana association and asked them to share their opinions on the changes that ARVs did or did not bring about. They admired the medicine for allowing so many people to get up and work for their survival. One thanked the late Prime Minister Meles Zenawi for making ARVs available.^20^ “The drug’s benefits are beyond description,” expressed another. “We have to take them with love,” maintained a woman who served as a Fana volunteer.

However, they also experienced continued hardships in life. “Are problems gone? They are there, on the people,” said a female member—“Some take their medicines, coming home at night after spending the day carrying soil and stones.^21^ To live, they have to work, eat, and swallow the medicines.” Another member who worked as a *telaleki* (messenger) in town said that after walking around the whole day, she often felt a burning sensation in her legs and wondered if it was a side effect of the drugs. The female volunteer said that each of her clients faced different kinds of difficulties and that it was difficult to listen to them. “Some can endure it; others not,” she posited.

Several years after the group meeting, Meseret informed me that she had left Fana’s office. She emphasized that it was her own choice to live her life. However, I suppose that the systematic indifference toward her care work with Fana members obliged her to choose between the association and “her own life,” though both were equally earnest threads in her story of moral survival. The ART scale-up in Africa gave a chance for survival to millions of individuals, including Meseret. However, it was through her engagements with the Fana members that she reclaimed a moral life that involved “negotiating important relations with others, doing work that means something to us, and living in some particular local place where others are also passionately engaged in these same existential activities” (Kleinman 2006, p. 2).

## Conclusion

This article presented some aspects of the multiple actions and engagements that formed and transformed HIV care in Ethiopia. Some emerged in the form of a praying multitude in the sacred mountain or a small self-help group in the quarter of Addis Ababa, each offering refuge to and forging a sense of solidarity among sufferers. Moreover, as ART scale-up took hold in Ethiopia, survivors of the epidemic, including Meseret, were invited to an emerging form of therapeutic citizenship, disposed to replace the silent and alienated community of sufferers. This process significantly altered the course of Meseret’s life. Among the many despairing women who lost their husbands to AIDS, she reestablished her life as a young leader in the network of HIV movements.

However, during the following years, Meseret found her association increasingly marginalized and defunded within the “pharmaceutically-centered model of public health,” which emerged as a byproduct of the ART scale-up (Biehl 2007, p. 1119). Thus, her story represents another case in which “the focus on keeping bodies alive with medicines may leave persons more vulnerable” when pharmaceutical interventions “fill in for a politics that can address socioeconomic inequalities and pursue a political program of change” (Prince 2012, p. 549). “Some people say that living with HIV is not a big deal. I think they are wrong. Once you are involved in [the life of each person], you see how complicated it is,” said Meseret, as she described the suffering that some of her association members experienced.

Surviving the HIV epidemic takes a mesh of care practices driven by needs and filled with meanings. Biomedical care, while vital, may not replace them or dominate the entire system. Instead, pharmaceuticals may become essential threads woven into the mesh of practices. Thus, my argument does not suggest that contemporary technologies for controlling a pandemic plainly and inevitably undermine the local forms of care and meanings of life they convey. How HIV care is conducted is “a function of the particular material–social assemblages in which care is enacted,” despite the virtual singularity of the mainstream intervention framework (Rhodes et al., 2019, p. 3). The experiences of surviving the HIV epidemic in Ethiopia, particularly the stories regarding the ways individuals like Meseret sought cures for their excessive suffering and how they were eventually caught up in the sphere of institutionalized indifference, provide a vital premise upon which claims for meaningful care are made, and ways to otherwise develop healthcare actions and engagements are sought.

## Footnotes

1 The term has been used, broadly, in discussions that relate the use of pharmacies with a range of issues, including cultural politics of health control (Whyte et al. 2009), biomedicalism promoted by pharmaceutical innovation (Abraham 2010), and capitalization of healthcare (Gaudilliere and Sunder Rajan 2021).

2 I use her official name with her consent and based on the fact that she represented an association of HIV-positive people, making her name and HIV status public. In what follows, the people representing the HIV movement in Ethiopia are also addressed with their official names, while other HIV-positive individuals are addressed with pseudonyms.

3 I conducted my research as a visiting researcher at the Institute of Ethiopian Studies, Addis Ababa University, which accepted my research proposal to conduct ethnographic research upon the HIV epidemic in the country.

4 Initially, patients with compromised immune systems (CD4 count less than 200 cells/mm^3^) were eligible for the national ART program. However, the clinical criterion was revised in 2012 to include patients with CD4 count less than 350 cells/mm^3^ and in 2014 to include all adults with CD4 count less than 500 cells/mm^3^, adults with active TB disease, hepatitis co-infection, pregnant and lactating women, and children less than 15 years of age (Assefa et al. 2017). Since 2016, all individuals diagnosed with HIV have been eligible for the free ART service regardless of their CD4 counts. As per the national guidelines, rapid ART initiation is offered to all individuals within seven days of a confirmed HIV diagnosis (FMoH 2018).

5 Data obtained from the World Bank Data, https://data.worldbank.org/indicator/SH.HIV.ARTC.ZS?locations=ET (accessed November 6, 2021).

6 In Ethiopia between 2005 and 2020, AIDS-related deaths decreased from 64,000 to 13,000, and HIV incidence decreased from 0.66 to 0.12 per 1,000 population. Data obtained from UNAIDS AIDSInfo, https://aidsinfo.unaids.org/ (accessed November 15, 2021).

7 In 2019, 85% of total ART spending in Ethiopia was financed by the Global Fund. Data obtained from UNAIDS HIV Financial Dashboard, https://hivfinancial.unaids.org/hivfinancialdashboards.html (accessed November 16, 2021).

8 By November 2021, the Global Fund had disbursed 2.59 billion USD to Ethiopia, which was the third-largest amount after Nigeria (2.88 billion USD) and Tanzania (2.64 billion USD). Data obtained from the Global Fund Data Service, https://data-service.theglobalfund.org/downloads (accessed November 5, 2021).

9 Other treatment and care spending included outpatient care services (12%), laboratory monitoring (11%), and provider-initiated testing and counseling (9%). HIV expenditures outside the treatment and care category included 79 million USD on prevention activities, 120 million on national system strengthening and program coordination, and 10 million on social protection services such as income generation activities (HAPCO 2013). Limited data were available concerning the spending trends during the following years. Another data set shows that total HIV spending in Ethiopia decreased by half between 2011 and 2019. In 2019, 52 million USD or 44% of HIV treatment, care, and support spending was on ART. HBC expenditure is not indicated. Data obtained from UNAIDS HIV Financial Dashboard, https://hivfinancial.unaids.org/hivfinancialdashboards.html (accessed November 16, 2021). Note that spending categories are inconsistent among the data sets.

10 Articles 2–2, 15–2, and 15–5 of the Charities and Societies Proclamation (FDRE 2009) stipulated that NGOs engaged in advocacy activities should not receive more than 10% of their revenues from foreign sources.

11 One of the factors that triggered such a shift in government policy toward NGOs was the general election in 2005,where the EPRDF, then the ruling party of Ethiopia, lost a considerable portion of parliament seats to the opposition parties. EPRDF leaders suspected that then proliferating NGOs, virtually all of which were financially supported by foreign humanitarian agencies, collectively functioned as a political constituency for the opposition parties.

12 Between 1998 and 2000, Ethiopia and Eritrea were engaged in a border conflict that is believed to have taken some 100,000 lives on both sides (ICG 2003).

13 However, since ART became widely available in Ethiopia, the use of holy water has been contested by health experts who identify it as a barrier against ART enrolment and retention (Wubshet et al. 2013; Kloos et al. 2013). Some followers of Ethiopian Orthodox resisted taking ARVs, claiming that they would compromise the power of holy water, while others accepted both (Berhanu 2010). Local policymakers, often Orthodox followers, preferred to address the issue by promoting ART *and* holy water rather than arguing against the latter. WHO Ethiopia Office worked with the Ministry of Health and the Ethiopian Orthodox Church to protect holy springs from being contaminated by the bacteria that could cause acute watery diarrhea or other waterborne diseases (WHO Africa 2017).

14 Data extracted from the Global Fund Data Explorer, https://data.theglobalfund.org/investments/grant/ETH-708-G07-H/1/ETH (accessed January 31, 2020).

15 The disproportionately higher number of females among Fana members may be partly explained by the higher HIV prevalence among women in Ethiopia. According to the Demographic and Health Survey conducted in 2011, the prevalence of HIV was 5.2% among urban females, whereas 2.9% among urban males (CSA 2012).

16 I could find no statistical data or estimates of the number of HIV-positive people in Welkite. My assumption was based on available data and knowledge. That is, the adult (15–49-year-old) population of Welkite was 17,496 in 2007 (CSA 2010), and it has rapidly increased ever since. HIV prevalence among the urban adult population of Ethiopia was estimated to be 4.1% in 2011 (CSA 2012) and has remained relatively stable.

17 Systematic analyses indicate that HIV prevalence is higher among the wealthier portion of the Ethiopian population (Lakew et al. 2015; Ejigu and Tadesse 2018), despite arguments that associate poverty with HIV risk in the country (Kloos and Mariam 2000; Sori 2012). These findings do not necessarily contradict each other because there are specific conditions in which deprivation leads to increased exposure to HIV and other health hazards. For example, Alene et al. (2019) found a higher prevalence of HIV and tuberculosis co-infection in less wealthy areas of the country.

18 They were typically motivated by religious or fraternal moral values that upheld sacrifice and devotion. The donor agencies justified using unpaid volunteers by arguing that the unpaid workforce would ensure the sustainability of the health services even after the external fundings were terminated (Maes 2016).

19 As Maes noted, the Ethiopian government moved against the use of unpaid community health workers in the first decade of the twenty-first century when it started to deploy health extension workers (HEWs) who were paid government employees. While HEWs contributed to improving the health of the population, their function was virtually limited to conveying a predetermined health information package (Bilal 2012). As a result, responding to the care needs of individual patients remained with unpaid volunteers (Maes 2016).

20 Meles served as the Prime Minister of Ethiopia from 1995 until his death in August 2012. His contribution to the ART expansion in Ethiopia is unclear to me, though clearly, it could not have come to fruition without his endorsement. His name was mentioned in the group discussion probably because they remembered him as the charismatic leader of the country.

21 They worked as day laborers at construction sites to make a living.
